# Development and Application of a Triplex qPCR Assay for the Simultaneous Detection of African Swine Fever Virus, Porcine Circovirus Type 2, and Porcine Circovirus Type 3

**DOI:** 10.3390/vetsci13090889

**Published:** 2026-08-31

**Authors:** Shunying Huang, Xing Chang, Jin Cui, Chunju Liu, Yanli Zou, Weijie Ren, Xiuju Han, Shan Liu, Xiaojing Yu, Yunxia Wang, Jinming Li, Zhiliang Wang, Jianchen Song

**Affiliations:** 1 Faculty of Agriculture, Yanbian University, Yanji 133002, China; huangsy3050@163.com; 2 China Animal Health and Epidemiology Center, Qingdao 266032, China; changxing@cahec.cn (X.C.); cuijin@cahec.cn (J.C.); liuchunju@cahec.cn (C.L.); zouyanli@cahec.cn (Y.Z.); renweijie@cahec.cn (W.R.); hanxiuju@cahec.cn (X.H.); liushan@cahec.cn (S.L.); yuxiaojing@cahec.cn (X.Y.); wangyunxia@cahec.cn (Y.W.); lijinming@cahec.cn (J.L.); 3 Reference Laboratory for African Swine Fever, Key Laboratory of Ministry of Agriculture and Rural Affairs (MARA), Qingdao 266032, China; 4 Qingdao Key Laboratory of Modern Bioengineering and Animal Disease Research, Qingdao 266032, China

**Keywords:** African swine fever virus, porcine circovirus type 2, porcine circovirus type 3, multiplex real-time PCR, molecular detection

## Abstract

African swine fever virus (ASFV), porcine circovirus type 2 (PCV2), and porcine circovirus type 3 (PCV3) represent important viral agents that threaten pig health. To date, no triplex real-time quantitative PCR (qPCR) method has been described for the concurrent identification of all three viruses. In this study, a triplex qPCR system was constructed using the ASFV B646L gene, PCV2 ORF1 gene, and PCV3 ORF1 gene as amplification targets, enabling simultaneous detection of ASFV, PCV2, and PCV3. The standard curves generated for the three viral targets all yielded R^2^ values of 0.999. The detection thresholds for ASFV, PCV2, and PCV3 were 10 copies/μL. Specificity testing showed that the assay amplified only the three intended viral targets and did not cross-react with other prevalent swine pathogens. Intra-assay and inter-assay reproducibility analyses yielded coefficients of variation below 2%. Seventy-nine clinical samples were tested using the established triplex qPCR assay and a previously published reference method. Positive and negative results were identical between the two assays, giving a concordance rate of 100%. These findings indicate that the triplex real-time qPCR assay provides a sensitive, dependable, and specific method for detecting ASFV, PCV2, and PCV3 infections and may facilitate epidemiological surveillance, as well as pathogen prevention and control.

## Simple Summary

African swine fever virus (ASFV), porcine circovirus type 2 (PCV2), and porcine circovirus type 3 (PCV3) have spread across numerous regions worldwide. Mixed infections involving these pathogens are now detected with increasing frequency in clinical practice and have imposed serious financial burdens on pig production. In the present work, a TaqMan probe-based triplex real-time quantitative PCR (qPCR) method was designed to identify ASFV, PCV2, and PCV3 in a single reaction. The newly developed assay exhibited strong analytical specificity, high detection sensitivity, and stable repeatability, indicating its practical value for clinical testing, epidemiological monitoring, and the prevention and management of these viral diseases.

## 1. Introduction

African swine fever (ASF) is an extremely contagious and serious infectious disease that affects both domestic and feral pigs. Among all the different varieties of pigs and their ages, there is a general prevalence of the susceptibility to infection with ASFV (African swine fever virus). Case fatality rates of the infection are extremely high, as they may reach 100% in some outbreaks [1]. Because of its fast transmission, wide range of hosts in swine species, and deadly clinical outcomes, ASF has become one of the most serious diseases affecting pig industry worldwide. The disease was first identified in Kenya in 1910 [2]. For a long time after that, reports about the presence of ASF were almost exclusively related to Africa. However, the situation changed drastically when ASFV spread to Georgia in 2007. After that, the virus managed to spread across several Eastern European countries [3]. Over the subsequent period, there were reports of ASF outbreaks in Europe, Africa, and Asia, causing considerable economic impacts on the international pig industry. The initial detection of ASF in China took place in 2018, following which the virus spread quickly across the country, posing considerable challenges for the Chinese pig industry [4].

Porcine circovirus (PCV) is a non-enveloped, tiny virus with a circular single-stranded DNA genome. Due to its minimal genome size and basic structure of its virion, PCV has been considered as one of the smallest animal viruses to be described to date [5]. The role of PCV infection in swine healthcare management has received much attention in recent years, since a number of PCV species are responsible for clinically important diseases in pigs. To date, there have been five species of PCV identified: PCV1, PCV2, PCV3, PCV4, and PCV5 [6,7,8]. Of these, PCV2 and PCV3 species are the most common circulating strains found in China; therefore, they are the most clinically relevant species in pig farming. PCV2 is closely associated with porcine circovirus-associated disease (PCVAD), a disease complex that can present with multiple clinical and pathological manifestations. On the other hand, infection by PCV2 in natural settings rarely happens as a singular phenomenon. Usually, it is observed along with the presence of other bacteria and viruses, which might cause the deterioration of the disease symptoms and enlargement of the disease spectrum [9]. PCV2-associated diseases include postweaning multisystemic wasting syndrome (PMWS) [10], porcine dermatitis and nephropathy syndrome (PDNS) [11], porcine respiratory disease complex (PRDC) [12], granulomatous enteritis, and reproductive disorders [13]. These diseases can occur in pigs of different developmental periods and can lead to low productivity, low reproductive efficiency, morbidity and difficulties in herd management. Thus, PCVAD causes serious reduction in productivity of pig farming and brings substantial financial losses to the swine industry. Unlike PCV2, PCV3 is a new virus that belongs to the family of porcine circoviruses. This virus was discovered in 2016 [8] and still does not have sufficient biological description and information on its pathogenesis. Despite the lack of understanding of the influence of PCV3 on disease development, recent findings show that PCV3 infection can be connected with some clinical syndromes, which include PDNS, reproductive disorders [14], and cardiac and multisystemic inflammation [8]. Clinical relevance of PCV3 in diseased subjects has been inferred in cases where there was systemic inflammation, reproductive disorders, and lesion profile similar to other PCV diseases. Co-infections of PCV2 and PCV3 have become common occurrences in recent years, where both viruses were found together in clinical samples from diseased subjects [15,16]. Co-detection of PCV2 and PCV3 in the same subject implies that both viruses might circulate in the pig population. Such a case poses a problem to clinicians, since clinical manifestations seen are not due to infection by one pathogen only. Moreover, co-infections make it difficult to understand the cause of the disease and evaluate the role played by each virus in disease development. Therefore, the co-detection of PCV2 and PCV3 is necessary in order to diagnose the disease and understand the epidemiology of PCV infections in pig farms. Another relevant consideration is the possible influence of PCV infections on the immune responses of the hosts. PCV2 or PCV3 infection may lead to inhibition of immune responses, which will weaken the defense mechanism of the host and increase its susceptibility to other pathogens [9,17]. In a case of secondary infection, the condition will become more severe, and treatments may not be very effective. Apart from co-infection between PCV2 and PCV3 infections, there are reports of co-infections of PCV2 with other pathogens, as well [18]. All these observations indicate that the diseases caused by PCVs do not happen in isolation, but as mixed infections. Therefore, techniques for the detection of multiple pathogens are necessary for the early detection and prevention of PCV diseases.

The efficiency, speed of amplification and high throughput of multiplex qPCR led to the extensive use of this technique in pathogen detection and established multiplex qPCR as an important method for molecular diagnostics of animal infectious diseases [19,20]. Unlike single-target analysis, multiplex qPCR provides the possibility of detecting several pathogens in one test. This advantage is especially valuable in the clinical diagnosis of swine diseases, in which different pathogens could cause the same clinical symptoms or be a part of co-infections. Thus, the implementation of multiplex qPCR could improve the efficiency of diagnosing, reduce the need for repeated analyses and allow quicker response for disease control. The ASFV, PCV2 and PCV3 are significant viral pathogens causing infections in pigs. Their concomitant occurrence in swine herds creates problems for clinical diagnosis and epidemiological monitoring. While the PCR-based techniques for the differentiation of ASFV, PCV2 and PCV3 have already been described, the triplex qPCR for their differential detection has not been developed yet [21]. This restriction can affect the efficiency of testing, especially where high volumes of clinical samples or multiple infections are concerned. To address this testing requirement, a triplex qPCR test was developed in the current research study in order to be able to simultaneously detect and discriminate between ASFV, PCV2 and PCV3. Through this technique, where all three viruses have been brought together in one reaction system, it is easier to identify both individual and mixed infections caused by these viruses. The test can be applied practically for diagnosis and prevention of ASFV, PCV2 and PCV3 infections in pigs.

## 2. Materials and Methods

### 2.1. Viral Nucleic Acids and Clinical Samples

The DNA or RNA genomes of ASFV, PCV2, PCV3, Porcine Delta coronavirus (PDCoV), Foot-and-mouth Disease Virus (FMDV), Getah Virus (GETV), Classical Swine Fever Virus (CSFV), Porcine Reproductive and Respiratory Syndrome Virus (PRRSV), Japanese Encephalitis Virus (JEV), Transmissible Gastroenteritis Virus (TGEV), and Porcine Epidemic Diarrhea Virus (PEDV) were maintained at the China Animal Health and Epidemiology Center (CAHEC). A total of 79 clinical specimens, including oral swabs, spleen tissues, and anticoagulated blood samples, were obtained from CAHEC and stored at −20 °C until further analysis. Thirteen ASFV-positive nucleic acid preparations and 50 clinical specimens were retrieved from the CAHEC repository. Because clinical specimens from naturally occurring mixed infections involving ASFV were unavailable, 16 simulated mixed-infection samples were prepared by combining ASFV-positive nucleic acids with PCV2- and/or PCV3-positive nucleic acids. These included eight samples simulating dual ASFV/PCV2 infection, five simulating dual ASFV/PCV3 infection, and three simulating triple ASFV/PCV2/PCV3 infection.

### 2.2. Extraction of Viral Nucleic Acids

One gram of tissue sample was ground and suspended in one milliliter of phosphate-buffered saline (PBS) in a grinding tube. Before centrifugation, the suspension was thoroughly mixed using a 3D Cryogenic Grinder (Shanghai Jingxin Industrial Development Co., Ltd., Shanghai, China). Blood samples were mixed completely before nucleic acid extraction, whereas swab samples were diluted in PBS prior to processing. The Purifier HT Automatic Nucleic Acid Extractor and the Finemag Rapid Magnetic Bead Virus DNA/RNA Extraction Kit, both provided by Jifan Biotechnology Co., Ltd. of Changzhou, China, were used to extract total nucleic acids from 200 μL of each processed sample.

### 2.3. Design of Primers and Probes

Reference sequences corresponding to the ASFV B646L gene, PCV2 ORF1 gene, and PCV3 ORF1 gene were downloaded from the GenBank database. SnapGene 4.3.6 was used to screen regions that were both virus-specific and highly conserved. Primer and probe sets were subsequently designed with Oligo 7.60, as listed in Table 1. The probes specific for ASFV B646L, PCV2 ORF1, and PCV3 ORF1 were labeled at the 5′ end with Cy5, FAM, and VIC, respectively. Primer and probe synthesis was performed by Sangon Biotech Co., Ltd. (Shanghai, China).

### 2.4. Preparation of Standard Plasmids

A recombinant pUC57 plasmid carrying partial fragments of the ASFV B646L, PCV2 ORF1, and PCV3 ORF1 genes was commercially synthesized by Tsingke Biotechnology Co., Ltd. (Beijing, China). The inserted sequences were verified by sequencing, and the confirmed plasmid was named pUC57-ASFV-PCV2-PCV3.

### 2.5. Optimization of Reaction Conditions

Triplex qPCR conditions were optimized using an ABI QuantStudio 5 qPCR system (Thermo Fisher Scientific Co., Ltd., Shanghai, China). Each 25.0 μL reaction contained 12.5 μL of 2× Animal Detection U+ Probe qPCR Super PreMix (Nanjing Vazyme Biotech Co., Ltd., Nanjing, China), 3.0 μL of DNA template, RNase-free water, and different concentrations of primers and probes. The final concentrations tested were 0.08–0.40 μM for each primer and 0.04–0.20 μM for each probe. The thermal program consisted of 2 min at 50 °C and 5 min at 95 °C, followed by 45 cycles of denaturation at 95 °C for 15 s and annealing at 56–61 °C for 1 min. Fluorescence signals were collected at the end of each cycle.

### 2.6. Establishment of Standard Curves

Standard plasmids diluted in a series of steps ranging from 1 × 10^8^ to 10 copies/μL were utilized as amplification templates once the best reaction components and cycle settings were identified. We conducted duplicate experiments for each dilution. Plasmid copy counts were plotted against their matching Cq values to generate standard curves, and the correlation coefficients (R^2^) were then computed. To find the amplification efficiency (*E*), we used the formula *E* = 10^(−1/*k*)^ − 1, where *k* is the standard curve slope.

### 2.7. Validation of Sensitivity

The established triplex qPCR assay’s analytical sensitivity was measured using standard plasmids diluted from 1 × 10^8^ to 1 × 10^−2^ copies/μL. For every concentration of plasmid, eight independent reactions were carried out. The limit of detection (LOD) was determined as the lowest concentration that resulted in positive amplification across all eight experiments. Clinical sensitivity was further evaluated using nucleic acids extracted from clinical blood samples positive for ASFV, PCV2, or PCV3, with one sample tested for each pathogen. Following absolute quantification by digital PCR (dPCR), each sample was diluted to the LOD concentration with RNase-free water and tested in eight independent replicate reactions. The detection rate for each virus was then calculated.

### 2.8. Validation of Specificity

Using nucleic acids from PDCoV, FMDV, GETV, ASFV, PCV2, PCV3, CSFV, PRRSV, JEV, TGEV, and PEDV as templates, the specificity of the proposed primers and probes was tested. Pigs that tested negative for viruses also had genomic DNA taken from their lymph nodes and spleen. One group served as positive controls using the usual plasmid, while the other group served as negative controls using RNase-free water.

### 2.9. Validation of Repeatability

We chose three different doses of the standard plasmid 1 × 10^6^, 1 × 10^4^, and 1 × 10^2^ copies/μL, as templates to find out how repeatable the experiment was. Each concentration was examined in three technical replicates within the same run to ensure intra-assay reproducibility. Inter-assay reproducibility was assessed in three independent experiments performed on different days by different operators using independently prepared batches of PCR mix. We computed the coefficients of variation (CVs) from the Cq values obtained from the intra- and inter-assay trials.

### 2.10. Detection of Clinical Samples

The established triplex qPCR test was used to assess the 79 clinical specimens kept in CAHEC. Previously published singleplex qPCR assays were used as reference methods for comparative evaluation. In addition to the PCV3 detection technique described by Zhang et al. [22], these specimens were also evaluated using the Chinese national standard methods for ASFV and PCV2 detection, GB/T 18648-2020 [23] and GB/T 21674-2025 [24], respectively. The diagnostic utility of the triplex qPCR assay for clinical samples was evaluated by comparing the outcomes acquired from different approaches.

## 3. Results

### 3.1. Construction of the Standard Plasmid

Sequencing verification demonstrated that the synthesized standard plasmid carried the anticipated 437 bp target fragment, and no nucleotide substitutions, insertions, or deletions were detected. The absorbance values of the plasmid at 260 and 280 nm were determined using a NanoDrop spectrophotometer (Thermo Fisher Scientific Co., Ltd., Shanghai, China). The quantified plasmid was then subjected to serial 10-fold dilution, producing standards with concentrations from 1 × 10^8^ to 1 × 10^−2^ copies/μL.

### 3.2. Determination of the Optimal Reaction Conditions

We found the best primer and probe concentrations, as well as the annealing temperature, by adjusting each experimental variable independently. Table 2 shows that, after optimization, the primer and probe concentrations were 0.32 μM and 0.20 μM, respectively, and the final reaction volume was 25 μL. An ideal thermal cycling program would begin at 50 °C for 2 min and 95 °C for 5 min, then go through 45 cycles of denaturation at 95 °C for 15 s and annealing/extension at 60 °C for 1 min. At the conclusion of every amplification cycle, fluorescence acquisition was carried out.

### 3.3. Generation of Standard Curves

**Figure 1:**
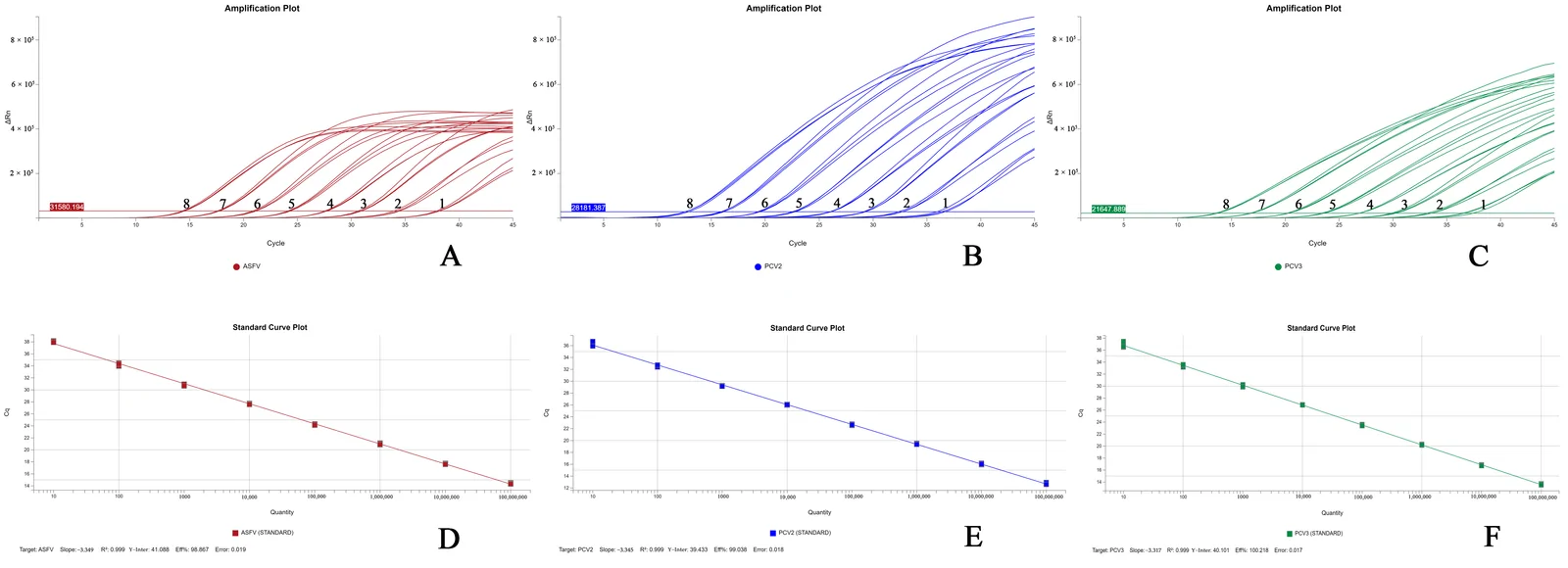
Standard curves of the triplex qPCR. The amplification curves for ASFV B646L gene (**A**), PCV2 ORF1 gene (**B**), and PCV3 ORF1 gene (**C**). The standard curves for ASFV B646L gene (**D**), PCV2 ORF1 gene (**E**), and PCV3 ORF1 gene (**F**). Note: 1–8: the final concentrations of the standard plasmid ranged from 1 × 10^1^ to 1 × 10^8^ copies/μL.

Under the optimized reaction conditions, plasmid standards at concentrations ranging from 1 × 10^8^ to 1 × 10^1^ copies/μL were amplified to generate standard curves for the triplex qPCR assay. The regression equations, correlation coefficients (R^2^), and amplification efficiencies were as follows: ASFV, *y* = −3.349*x* + 41.088, 0.999, and 98.867%; PCV2, *y* = −3.345*x* + 39.433, 0.999, and 99.038%; and PCV3, *y* = −3.317*x* + 40.101, 0.999, and 100.218% (Figure 1). Because all R^2^ values exceeded 0.99, the assay showed strong linearity across the tested concentration range.

### 3.4. Sensitivity of the Triplex qPCR Assay

Serially diluted standard plasmids were used as templates to determine the sensitivity of the triplex qPCR assay. For each plasmid concentration, eight replicate reactions were conducted to calculate the positive detection rate. All duplicates tested positive for ASFV, PCV2, and PCV3 at quantities ranging from 1 × 10^8^ to 1 × 10^1^ copies/μL (Table 3), The rates of detection dropped when the concentration of plasmid was less than 1 × 10^1^ copies/μL. When all eight duplicates came back positive at the same plasmid concentration, we said that this was the LOD. The established triplex qPCR test demonstrated great analytical sensitivity with a limit of detection (LOD) of 10 copies/μL for ASFV, PCV2, and PCV3, according to this criterion. At the LOD, the mean Cq values were 35.18 ± 0.33 for ASFV, 35.45 ± 0.51 for PCV2, and 35.54 ± 0.59 for PCV3 (mean ± SD, *n* = 8). Samples were classified as positive when Cq ≤ 36 with a characteristic amplification curve, negative when no Cq value was obtained or when Cq ≥ 40 without a characteristic amplification curve, and equivocal when 36 < Cq < 40 with a characteristic amplification curve. Equivocal samples were retested using twice the template volume (6 μL) and were reclassified as positive if the repeat test yielded Cq < 40 with a characteristic amplification curve; otherwise, they were classified as negative. The same interpretation criteria were applied to all three targets.

Clinical sensitivity testing showed that, at a concentration of 10 copies/µL, all eight replicate reactions performed with nucleic acids from ASFV-, PCV2-, and PCV3-positive clinical blood samples yielded positive results, corresponding to a detection rate of 100% for each virus. These findings indicate that the assay retained high sensitivity for low-concentration targets in clinical sample matrices.

### 3.5. Specificity of the Triplex qPCR Assay

**Figure 2:**
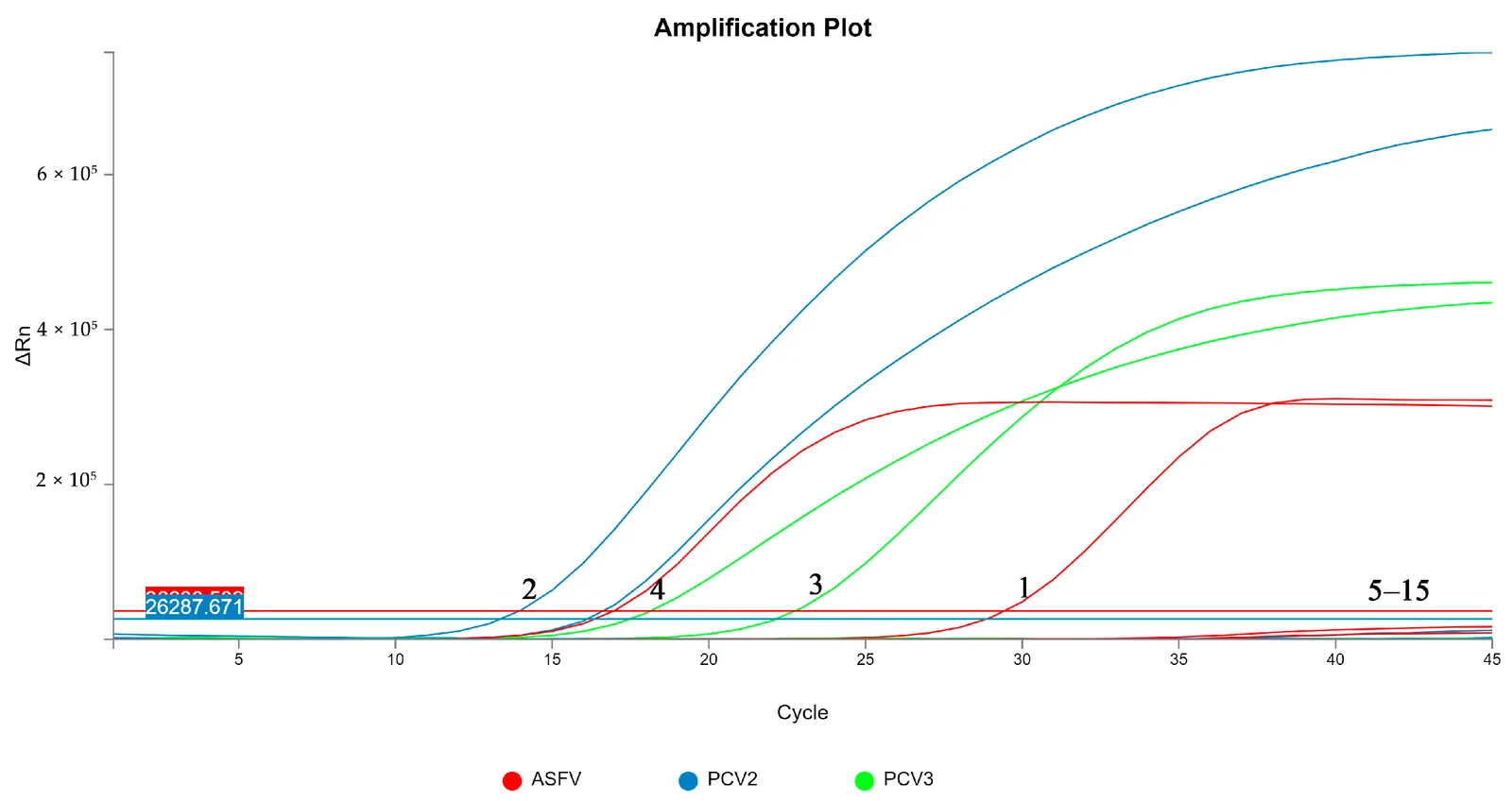
Specific amplification curves of the triplex qPCR: 1: ASFV; 2: PCV2; 3: PCV3; 4: pUC57-ASFV-PCV2-PCV3; 5–15: PDCoV, FMDV, GETV, CSFV, PRRSV, JEV, TGEV, PEDV, spleen, lymph node, and RNase-free water.

To examine assay specificity, nucleic acids extracted from PDCoV, FMDV, GETV, ASFV, PCV2, PCV3, CSFV, PRRSV, JEV, TGEV, and PEDV were used as amplification templates. The recombinant plasmid pUC57-ASFV-PCV2-PCV3, nucleic acids from spleen and lymph node tissues of virus-negative pigs, and RNase-free water were also included. The results showed that each target-positive viral nucleic acid produced only the expected fluorescence amplification signal, whereas pUC57-ASFV-PCV2-PCV3 generated three target-specific amplification curves. No amplification signal was detected for the non-target pathogens, virus-negative tissue samples, or RNase-free water control (Figure 2). These findings confirmed the high specificity of the developed triplex qPCR assay.

### 3.6. Repeatability Analysis of the Triplex qPCR Assay

Templates for repeatability testing were chosen from standard plasmids with concentrations of 1 × 10^6^, 1 × 10^4^, and 1 × 10^2^ copies/μL. To measure intra-assay variance, three replicate reactions were carried out inside the same experiment; inter-assay variation was assessed in three independent experiments conducted on different days by different operators using independently prepared batches of PCR mix. The test displayed favorable repeatability, as shown by the intra- and inter-assay coefficients of variation (CVs) being less than 2% (Table 4).

### 3.7. Detection Results for Clinical Samples

The clinical applicability of the established triplex qPCR assay was assessed using 79 clinical specimens. As shown in Table 5, the results were completely consistent with those obtained using the reference methods. Among the 79 specimens tested, single infections with ASFV, PCV2, and PCV3 were detected in 16.5% (13/79), 21.5% (17/79), and 12.7% (10/79) of samples, respectively. Co-infections with PCV2 + PCV3, ASFV + PCV2, ASFV + PCV3, and ASFV + PCV2 + PCV3 were detected in 6.3% (5/79), 10.1% (8/79), 6.3% (5/79), and 3.8% (3/79) of samples, respectively. The remaining 18 specimens (22.8%) were negative for all three pathogens. Overall concordance between the triplex qPCR assay and the reference assays was 100%, supporting the potential utility of the developed method for field surveillance and laboratory diagnosis.

## 4. Discussion

Since 2018, ASF has posed a serious threat to the sustainable and stable development of the swine industry in China, as well as to global pig production. It has also become a major animal disease prioritized for prevention and control [1,25]. PCV2 and PCV3 are widespread pathogens associated with multifactorial disease complexes and reproductive disorders in swine. PCV2 and PCV3 are often detected together and may cause immunosuppression, increasing the risk of co-infection. Previous studies have shown that ASFV can co-infect swine with PCV2 or PCV3 [26,27]. Whether infection with PCV2 or PCV3 increases the susceptibility of pigs to later ASFV infection has not been fully determined and warrants further study. Given the continued circulation of ASFV among pig populations in China, mixed infections involving ASFV and porcine circoviruses are likely to be detected more frequently. Thus, an efficient multiplex assay with rapid turnaround, high accuracy, and strong analytical sensitivity is needed.

PCR is widely used for pathogen detection because of its high specificity, sensitivity, and reliability. Multiplex PCR enables the simultaneous, rapid, and accurate detection of diverse pathogens. Compared with singleplex PCR, multiplex PCR maintains comparable sensitivity and specificity, while improving laboratory throughput and providing substantial economic benefits in time and resource management [20]. Numerous studies have developed independent PCR assays to detect ASFV, PCV2, and PCV3. PCR was applied to detect ASFV nucleic acid as early as the 1990s [28]. Several conventional multiplex PCR methods have been described. E et al. developed a triplex PCR method for identifying ASFV, PCV2, and PCV3 [21]. Although this method exhibited satisfactory specificity and repeatability, its analytical sensitivity was relatively low, with a detection limit of 10^4^ copies/μL.

With improvements in molecular diagnostic technology, qPCR has become a major approach for detecting viral nucleic acids. Fernández-Pinero et al. reported a real-time qPCR method targeting the ASFV VP72 gene, which markedly shortened the detection process [29]. Brunborg et al. established a PCV2 ORF2-based qPCR assay with a detection limit of 10 copies per sample [30]. Wang et al. developed a highly sensitive PCV3 ORF2-targeting assay, achieving a detection limit of 100 copies/μL [31].

More recently, multiplex qPCR has progressed rapidly and has allowed multiple pathogens to be identified simultaneously within a short time. Several multiplex qPCR systems have been developed for different combinations of ASFV, PCV2, and PCV3. For example, Li et al. established a triplex qPCR assay targeting the ASFV B646L, PRV gE, and PCV2 Rep genes for the simultaneous detection of ASFV, pseudorabies virus (PRV), and PCV2, achieving an LOD of 10 copies/µL [32]. Chen et al. subsequently developed a triplex qPCR assay for simultaneous detection of PCV2, PCV3, and PCV4 by targeting the PCV2 and PCV4 ORF1 genes, as well as the PCV3 ORF2 gene, with corresponding LODs of 53.3, 12.0, and 13.8 copies/µL, respectively [33]. A duplex qPCR assay for PCV2 and PCV3 has also been reported, using specific primers and probes targeting the PCV2 Cap and PCV3 Rep genes. This assay achieved LODs of 2.9 and 22.5 copies/µL for PCV2 and PCV3 plasmids, respectively [34]. Nevertheless, no single qPCR platform has been available for the simultaneous identification of ASFV, PCV2, and PCV3. This absence limits the efficiency of surveillance for co-infections in swine herds. To overcome this limitation, a new triplex qPCR assay was developed in the present study.

Selection of appropriate target genes is fundamental to PCR assay design because it directly affects analytical specificity and sensitivity. The ASFV B646L gene encodes the major capsid protein and is a principal target for ASFV genotyping [35,36]. Its high degree of sequence conservation also makes it well suited for molecular detection. PCV2 and PCV3 are small, non-enveloped DNA viruses with two major open reading frames (ORFs): ORF1 encodes the replication-associated protein (Rep), whereas ORF2 encodes the capsid protein (Cap), the sole structural protein [14,37]. Both the Cap and Rep genes are commonly used as molecular targets for differential diagnosis because of their high sequence conservation [38,39]. Accordingly, the ASFV B646L gene and ORF1 genes of PCV2 and PCV3 were selected as targets in the present study. Primers and probes were designed against highly conserved genomic regions to establish a triplex qPCR assay with high analytical specificity and sensitivity for simultaneous detection of the three pathogens. Nevertheless, ASFV, PCV2, and PCV3 exhibit considerable genetic diversity worldwide. Continuous surveillance of viral evolution and periodic reassessment and updating of primer and probe sequences will therefore be necessary to maintain reliable detection as new variants emerge.

In this study, three pairs of virus-specific primers and three matched probes were designed against conserved genomic regions of ASFV, PCV2, and PCV3. The established triplex qPCR assay allowed all three viruses to be detected in one reaction using a Taq DNA polymerase-containing PCR master mix. This format simplified the diagnostic workflow and decreased the possibility of amplicon contamination. Cy5, FAM, and VIC were used as reporter dyes, and no signal interference was detected among the three fluorescence channels. In addition, the three target fragments were cloned into a single plasmid vector, which maintained equivalent copy number proportions among the targets. This design reduced the cost of standard plasmid construction and lowered the systematic variation that may arise when three separate plasmids are added independently. The developed assay showed favorable analytical performance. The analytical LOD was 10 copies/μL for ASFV, PCV2, and PCV3, comparable to those reported for previously developed duplex and multiplex qPCR assays. Clinical sensitivity testing further showed that the assay retained a high detection capability for low-concentration targets in clinical sample matrices. No cross-amplification was observed with other porcine pathogens, including PDCoV, FMDV, GETV, CSFV, PRRSV, JEV, TGEV, and PEDV. Plasmid copy number logarithmic values showed a robust linear association with Cq values in the standard curves. Good repeatability was confirmed by intra- and inter-assay coefficients of variation being less than 2%. The assay that was developed here was also used to analyze 50 clinical specimens, along with the methods reported by Zhang et al. [22] and GB/T 18648-2020 [23] and GB/T 21674-2025 [24], which are reference methods for ASFV, PCV2, and PCV3 detection, respectively. A 100% concordance rate indicates that the findings are entirely consistent.

Although the developed triplex qPCR assay demonstrated robust analytical and diagnostic performance, several limitations should be acknowledged. First, the specificity panel did not include several genetically or clinically relevant pathogens, including PCV1, PCV4, PCV5, other circoviruses, porcine parvovirus, and swine influenza virus. Second, all clinical specimens and reference strains originated from China, and assay validation was therefore based on a geographically and genetically restricted panel. Future studies should expand the validation panel as additional reference strains become available. Third, because of the modest sample size (n = 79) and the lack of clinical specimens from naturally occurring ASFV + PCV3, ASFV + PCV2, and ASFV + PCV2 + PCV3 co-infections, dual- and triple-infection samples were simulated by artificially mixing pathogen-positive nucleic acids. The diagnostic performance of the assay in naturally occurring co-infections therefore requires further validation. In addition, although the assay enables rapid and sensitive detection of ASFV, PCV2, and PCV3, it does not discriminate among viral genotypes. Absolute quantification of in-dividual viral loads in co-infected specimens is also not possible; only relative abundance can be estimated from Cq values. Finally, the current assay does not include an internal control, primarily to minimize potential inter-target interference and preserve accurate detection of the three target pathogens. However, the absence of an internal control prevents direct monitoring of sample quality and PCR inhibition, and may therefore increase the risk of false-negative results. Future work should incorporate an internal control and systematically evaluate its effects on sensitivity, specificity, and inter-target interference to further improve assay robustness.

## 5. Conclusions

The triplex qPCR method established in this study meets the diagnostic demand for simultaneous detection of ASFV, PCV2, and PCV3 co-infections. As the first multiplex assay designed to cover these three viral pathogens together, it exhibited strong sensitivity, specificity, and repeatability. This method offers a useful technical tool for clinical diagnosis and provides support for epidemiological surveillance of swine viral diseases.

## Figures and Tables

**Table 1:** Designed primers and probes.

Primer/Probe	Sequence (5′ to 3′)	Targe Gene	Site (bp)
ASFV-F	GATATAGATGAACATGCGTCTGG	B646L	134
ASFV-R	CAAAATCCTCATCAACACCG		
ASFV-P	CY5-CGCCATTATGCAGCCCACTCACCAC-BHQ2		
PCV2-F	TGCATTTTCCCGCTCACT	ORF1	154
PCV2-R	CTAGATCTCAAGGACAACGG		
PCV2-P	FAM-AAATTTCTGACAAACGTTACAGGGT-BHQ1		
PCV3-F	TGCAAGAAAGAGGGGGAT	ORF1	130
PCV3-R	GCATCTTCTCCGCAACTTC		
PCV3-P	VIC-CCTCTTCGGGTACCAGATCGGATCT-BHQ1		

**Table 2:** Reaction system for the triplex qPCR assay.

Reagent	Volume (μL) or Concentration (μM)
2× Animal Detection U^+^ Probe qPCR Super PreMix	10 μL
ASFV-B646L-F (10 μM)	0.8 μL (0.32 μM)
ASFV-B646L-R (10 μM)	0.8 μL (0.32 μM)
ASFV-B646L-P (10 μM)	0.5 μL (0.2 μM)
PCV2-ORF1-F (10 μM)	0.8 μL (0.32 μM)
PCV2-ORF1-R (10 μM)	0.8 μL (0.32 μM)
PCV2-ORF1-P (10 μM)	0.5 μL (0.2 μM)
PCV3-ORF1-F (10 μM)	0.8 μL (0.32 μM)
PCV3-ORF1-R (10 μM)	0.8 μL (0.32 μM)
PCV3-ORF1-P (10 μM)	0.5 μL (0.2 μM)
Template	3 μL
RNase-free water	3.4 μL

**Table 3:** Detection rates at different standard plasmid concentrations.

Concentration (Copies/μL)	Detection Rate (%)		
	ASFV	PCV2	PCV3
1 × 10^8^–1 × 10^2^	100%	100%	100%
10	100%	100%	100%
1	75%	75%	75%
0.1	50%	37.5%	37.5%
0.01	12.5%	12.5%	12.5%

**Table 4:** Intra-assay and inter-assay reproducibility analysis of the triplex qPCR assay.

Virus	Concentration (Copies/μL)	Cq Values of Intra-Assay			Cq Values of Inter-Assay		
		$\bar{X}$	SD	CV (%)	$\bar{X}$	SD	CV (%)
ASFV	$10^{2}$	32.41	0.03	0.10%	32.40	0.20	0.63%
	$10^{4}$	26.80	0.09	0.34%	26.83	0.19	0.72%
	$10^{6}$	18.99	0.16	0.83%	18.98	0.15	0.78%
PCV2	$10^{2}$	31.43	0.05	0.15%	31.58	0.16	0.52%
	$10^{4}$	25.86	0.10	0.37%	26.08	0.25	0.96%
	$10^{6}$	17.83	0.19	1.08%	17.93	0.23	1.29%
PCV3	$10^{2}$	31.58	0.06	0.18%	31.72	0.18	0.58%
	$10^{4}$	25.95	0.06	0.23%	26.25	0.41	1.58%
	$10^{6}$	18.04	0.20	1.14%	18.12	0.17	0.93%

**Table 5:** Detection results for clinical samples.

Virus	Triplex qPCR		Reference Methods (Singleplex qPCR)		Matrix Distribution (n)
	Positive Number	Positive Detection Rates (%)	Positive Number	Positive Detection Rates (%)	
ASFV	13	16.5	13	16.5	swabs (3); Spleen (4); Blood (6)
PCV2	17	21.5	17	21.5	swabs (6); Spleen (6); Blood (5)
PCV3	10	12.7	10	12.7	swabs (1); Spleen (7); Blood (2)
ASFV + PCV2	8	10.1	8	10.1	mixed positive nucleic acids (8)
ASFV + PCV3	5	6.3	5	6.3	mixed positive nucleic acids (5)
PCV2 + PCV3	5	6.3	5	6.3	Swabs (1); Spleen (2); Blood (2)
ASFV + PCV2 + PCV3	3	3.8	3	3.8	mixed positive nucleic acids (3)

## Data Availability

The original contributions presented in this study are included in the article. Further inquiries can be directed to the corresponding authors.

## Abbreviations

The following abbreviations are used in this manuscript:

Cq	Quantification cycle
ΔRn	Delta-Normalized Reporter
$\bar{X}$	Mean value
SD	Standard deviation
